# Hippo Pathway Regulates Cell Proliferation in Skin Epidermis Exposed to Mechanical Forces

**DOI:** 10.1111/jcmm.70674

**Published:** 2025-06-27

**Authors:** Joanna K. Ledwon, Bianka Progri, Sarah A. Applebaum, Oveyaa Vignesh, Alice Yau, Angie H. Aguilar, Adrian B. Tepole, Arun K. Gosain

**Affiliations:** ^1^ Division of Plastic Surgery Northwestern University Feinberg School of Medicine Chicago Illinois USA; ^2^ Department of Plastic and Reconstructive Surgery Ann and Robert H. Lurie Children's Hospital of Chicago Stanley Manne Children's Research Institute Chicago Illinois USA; ^3^ Department of Mechanical Engineering Purdue University West Lafayette Indiana USA

**Keywords:** cell proliferation, epidermis, hippo pathway, mechanical forces, mechanotransduction, skin growth, tissue expansion

## Abstract

Tissue expansion is an integral component of reconstructive surgery used to promote native skin growth. This process is driven by the gradual inflation of the tissue expander placed subcutaneously on the patient's body. Despite its widespread use, the lack of in vivo evidence on the biological processes underlying skin growth has limited technological advancements. Here, we explore the gene and protein expression changes that control mechanically induced skin growth during tissue expansion. Using a porcine tissue expansion model, we revealed that skin expansion disrupts key components responsible for epithelial integrity, as evidenced by the loss of E‐cadherin and alpha‐catenin expression in expanded skin compared to the unexpanded control. This disruption correlates with the translocation of the transcriptional factor YAP1 from the membrane to the nucleus, activating keratinocyte proliferation and possibly regulating other critical processes involved in skin adaptation to mechanical stretch. Our data show that in vivo cell proliferation is mediated by force‐induced changes in the composition of molecular complexes formed by E‐cadherin, alpha‐catenin, and YAP1.

## 1 Introduction

Skin tissue expansion is a long‐established and effective surgical treatment utilised to promote growth of native skin during reconstructive surgery [1, 2, 3, 4]. This multi‐stage procedure starts with subcutaneous placement of tissue expanders, followed by periodic saline injections to fill the expanders over the course of weeks to months until the desired increase in skin surface area is achieved to proceed with reconstruction [5]. Hence, skin cells are subject to an increase in tensile forces that arise from the inflated tissue expander, activating unique ability of skin cells to proliferate in response to mechanical stimulation [5, 6, 7]. Tissue expansion is commonly used in adult and paediatric patients in various medical conditions, including burn deformities, craniofacial abnormalities, giant congenital nevi, and post‐mastectomy breast reconstruction [2, 3, 8, 9, 10].

Biomechanical response to mechanosensing allows cells to quickly adapt to the surrounding environment by engaging specific transcriptome programs and biochemical pathways [11, 12, 13, 14]. The E‐cadherin/Hippo/YAP1 pathway is thought to play a central role in regulating mechanotransduction that restricts proliferation in adult tissues [14, 15, 16, 17, 18]. E‐cadherin, a transmembrane protein that forms homotypic dimers between neighbouring cells, is a central component of adherens junctions (AJs) that mainly mediate cell adhesion. The AJs also regulate tissue architecture and its dynamics through the regulation of cell proliferation, survival, and migration [19]. In AJs, E‐cadherin binds beta‐catenin and alpha‐catenin to constitute the cadherin‐catenin complex [20]. The presence of E‐cadherin adhesions has been shown to negatively regulate cellular growth signals through stimulating the Hippo signalling pathway [21, 22]. Disturbance in E‐cadherin adhesions releases Yes‐associated protein 1 (YAP1), which is translocated to the nucleus, where it promotes cell cycle entry [22, 23, 24, 25, 26]. The role of YAP1 in stretch‐induced cell proliferation was demonstrated in vitro and in a murine model [16, 27, 28]. Other studies have demonstrated that YAP1 activity in keratinocytes is controlled by alpha‐catenin that sequesters YAP1 in the cytoplasm, acting as its upstream negative regulator [15, 29]. In the nucleus, YAP1 interacts with TEA domain (TEAD) transcription factors and acts as a transcriptional coactivator that activates target gene expression to restore tissue homoeostasis [30, 31, 32, 33].

Although our study and others have shown that tissue expansion induces basal keratinocyte proliferation in expanded skin [7, 34, 35], the underlying mechanisms that enable skin to grow under mechanical stimulation in vivo in large animal models that closely resemble humans have not been well described. Understanding the molecular mechanisms induced by tissue expansion is an essential step towards translational research focused on improving tissue regeneration in clinical settings. Herein, we explore the role of the E‐cadherin/Hippo/YAP1 pathway in mechanically induced basal keratinocyte proliferation using a porcine model of tissue expansion.

## 2 Materials and Methods

### 2.1 Animal Model and Study Design

The animals were housed in an animal facility at the Center for Comparative Medicine (Northwestern University, Chicago, IL, USA) accredited by the Association for Assessment and Accreditation of Laboratory Animal Care International. The study was conducted on a porcine model of tissue expansion, which is the closest non‐primate surrogate of tissue expansion in humans. Due to the structural and physiological similarities between porcine and human skin [36, 37], the porcine model serves as an excellent tool for studying the molecular response of the skin to tissue expansion. Our group has successfully utilised this model in previous studies [6, 7, 38, 39, 40]. Tissue expanders were surgically implanted subcutaneously beneath tattooed grids on the anterior site of each pig. To ensure even distribution of the expander within the dissected pocket, an initial 10 mL saline fill was administered at the time of implantation. Two weeks post‐implantation, the expanders were further injected with 60 mL of saline to initiate controlled mechanical skin expansion. Tissue samples were collected at day 1, day 3, and day 7 post‐injection to assess the dynamic changes in skin molecular response, followed by animal euthanasia. Additionally, unexpanded skin samples were obtained from each animal and used as controls to establish a baseline for comparison. Each experimental condition was tested in two biological repeats (Figure S1).

### 2.2 Total RNA Extraction

The full‐thickness skin biopsies preserved in All Protect Reagent (Qiagen) were homogenised using TissueLyser LT homogeniser (Qiagen) and 7 mm stainless‐steel beads (Qiagen) in three cycles for 10 min at 50,000 Hz. Total RNA was extracted using RNAeasy Mini Kit (Qiagen) with DNase I treatment (Qiagen) according to the manufacturer's recommendations. The tissue homogenisation and extraction procedures have previously been described in detail [38].

### 2.3 Quantitative Real‐Time PCR Analysis (qRT‐PCR)

The qRT‐PCR analysis was performed on QuantStudio 6 Flex Real‐Time PCR Systems (Applied Biosystems) using TaqMan Assay (Applied Biosystems), including TaqMan Fast Advanced Master Mix and commercially available TaqMan probe for *CDH1*, as previously described [41]. The cycle quantification (Cq) values were calculated using the threshold cycle method, and fold change of gene expression was calculated with the 2^‐ΔΔCT^ method [42]. The results were normalised to the geometric mean calculated for two reference genes: *RLPL0* (Ribosomal Protein Lateral Stalk Subunit P0) and *RPS27A* (Ribosomal Protein S27a).

### 2.4 Immunofluorescence (IF) Staining

IF staining was performed on 4 μm cross skin sections preserved in 10% formalin and embedded in paraffin according to the protocol described previously [6, 38]. The sections were incubated with the following primary antibodies: anti‐E‐cadherin (1:500; Proteintech, 20,874‐1‐AP), anti‐alpha‐Catenin (1;500; Fitzgerald, 70R‐49,611), anti‐YAP1 (1:100; Abcam, ab114862), and anti‐Ki‐67 (1:100; Invitrogen, 14–5698‐82) overnight at 4°C. Signal was detected using the following fluorochrome‐conjugated secondary antibodies incubated for 1 h at room temperature: donkey anti‐rabbit IgG Alexa Fluor 546 (1:200; Invitrogen, A10040), and goat anti‐rat IgG Alexa Fluor 647 (1:200; Invitrogen, A‐21247). Nuclei were stained using 1 μg/mL DAPI (Invitrogen). The specificity of the staining was tested by performing staining without the primary antibodies.

### 2.5 Confocal Imaging

IF stained slides were photographed on a Zeiss LSM 880 laser scanning confocal microscope using a 40× water immersion objective. The imaging conditions were determined for each target protein on control tissue and applied to the corresponding experimental tissue. The Zen Blue 3.5 software (Zeiss) was used to measure total and nuclear fluorescence intensity.

### 2.6 Fluorescence Intensity Quantification

Total fluorescence intensity (sum of pixel intensities) was quantified using the Zen Blue software and normalised to the number of nuclei. The quantitative analysis was performed on at least three non‐consecutive sections (*n* = 3) to account for sample‐dependent variability in two biological repeats (*n* = 2). Images collected from the experimental tissue and corresponding controls were analysed using the same value of the maximum threshold to objectively quantify signal intensity. The results are presented as the relative changes of total fluorescence signal intensity per cell between control and expanded skin. The mean value for control was set as 1. The total and nuclear fractions of fluorescent signal were examined for YAP1. Nuclei were defined based on DAPI staining.

### 2.7 Normal Human Skin Sections

Human skin tissue was collected from patients enrolled in the study STU00210182 after obtaining written informed consent. The study was approved by the Northwestern University Institutional Review Board.

### 2.8 Statistical Analysis

The statistical significance of the results was assessed using Student's unpaired *t*‐test. All data sets passed the Shapiro–Wilk normality test. Data for experimental groups were compared with corresponding unexpanded controls. Statistical analyses were performed using GraphPad Prism 9.2.0 software (GraphPad Software Inc.). *p* < 0.05 was considered significant.

## 3 Results

### 3.1 E‐Cadherin Expression Is Negatively Regulated in Skin by Mechanical Stretching

**FIGURE 1:**
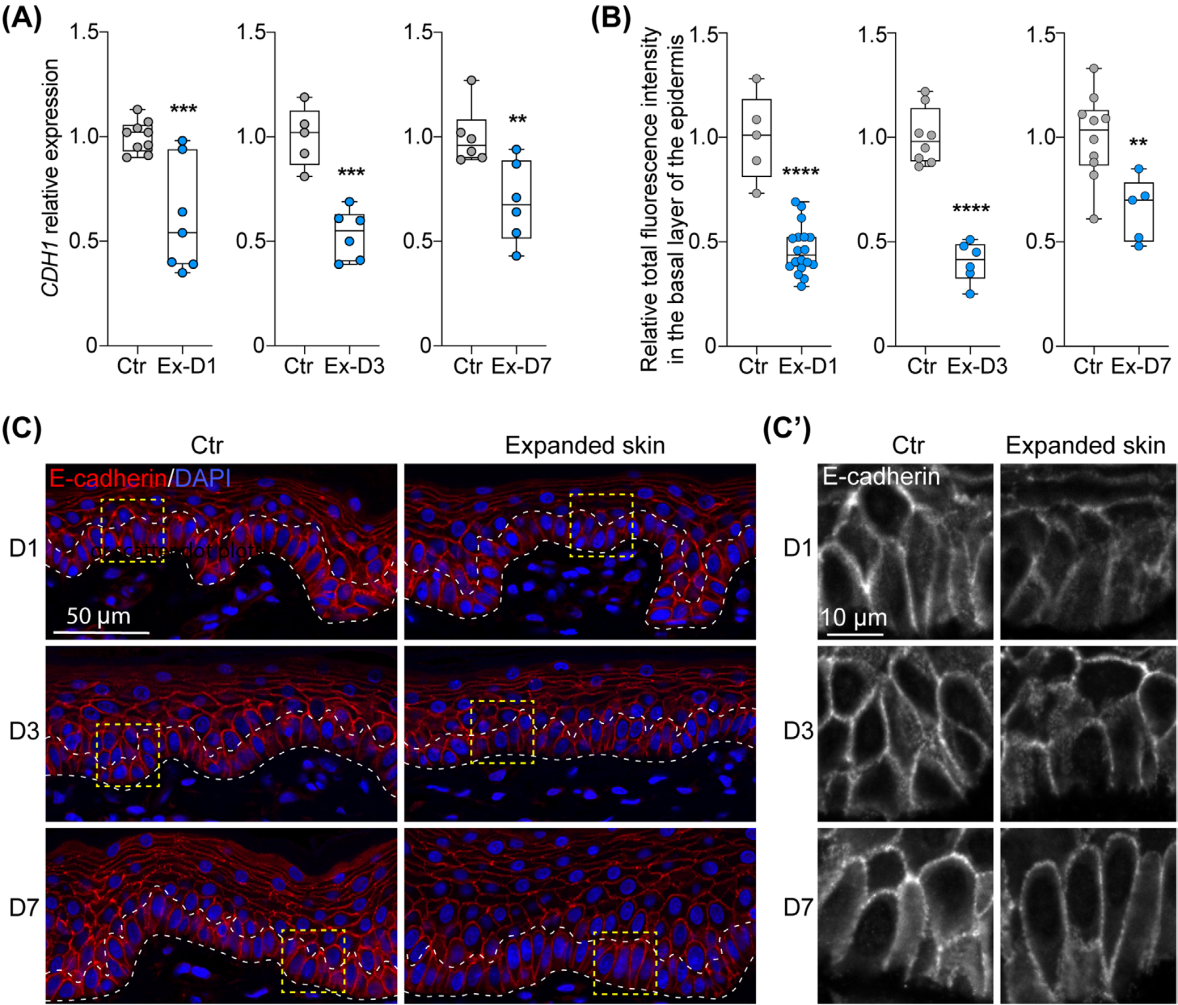
Changes in CDH1 expression in skin samples during tissue expansion. (A) Relative expression of *CDH1* expression assessed by qRT‐PCR in control (Ctr) and expanded skin (Ex) collected at day 1 (D1), day 3 (D3) and day 7 (D7) of expansion compared to corresponding unexpanded control. The values were normalised to the geometric mean calculated for two reference genes: *RLPL0* and *B2M*. Data on graphs present results for one of two tested biological repeats. Each dot represents results for one biopsy (*n* ≥ 5). (B) Quantitative analysis and **(C)** representative images (40× magnification) of IF staining of E‐cadherin expression in control (Ctr) and expanded skin (Ex) at day 1 (D1), day 3 (D3) and day 7 (D7) of expansion. In (B), each dot represents results for one picture. In (A) and (B), the line in the middle of the box is plotted at the median and the whiskers represent minimum and maximum values. Values represent fold changes between controls and tested samples, and the average value for each control was set as 1. In (C), dashed lines mark the basal layer of the epidermis. (C′) presents the magnified views of the area marked in panel C. Statistical significance calculated with unpaired Student *t*‐test is shown as **p ≤ 0.01, ***p ≤ 0.001, ****p ≤ 0.0001.

As Hippo signalling‐mediated mechanotransduction has been involved in cell proliferation and tissue growth in physiological and pathological conditions [43], we first asked how the expression of E‐cadherin, the upstream regulator of the Hippo signalling pathway, is affected in skin samples during tissue expansion. The evaluation of *CDH1* transcription on RNA extracted from full‐thickness skin biopsies using qRT‐PCR showed a significant decrease by an average of 36%, 53%, and 32% at day 1, day 3, and day 7 of expansion, respectively (Figure 1A presents numerical values for one of the biological repeat; Day 1: FC = 0.61, *p* = 0.0007; Day 3: FC = 0.53, *p* = 0.0002; Day 7: FC = 0.69, *p* = 0.0098). Downregulation of E‐cadherin expression in expanded skin was confirmed by immunofluorescence (IF) staining (Figure 1B,C,C'). Accordingly, E‐cadherin expression in the basal layer of the epidermis, measured as total fluorescence intensity normalised to the number of cells, was significantly reduced by an average of 44% as early as day 1 after expander inflation, reaching a maximum average decrease of 48% on day 3 of expansion compared to the corresponding unexpanded controls. By day 7 of expansion, the average decrease in E‐cadherin expression had tapered to 39%, but remained significantly lower compared to unexpanded skin (Figure 1B presents numerical values for the representative staining; Day 1: FC = 0.46, *p* < 0.0001; Day 3: FC = 0.40, *p* < 0.0001; Day 7: FC = 0.60, *p* = 0.0055). These results indicate that E‐cadherin expression diminishes significantly in the epidermis during tissue expansion in response to mechanical stretching, and its expression is still disturbed a week after expansion is initiated.

### 3.2 Alpha‐Catenin Expression Does Not Decline Immediately in Response to Mechanical Stretching

**FIGURE 2:**
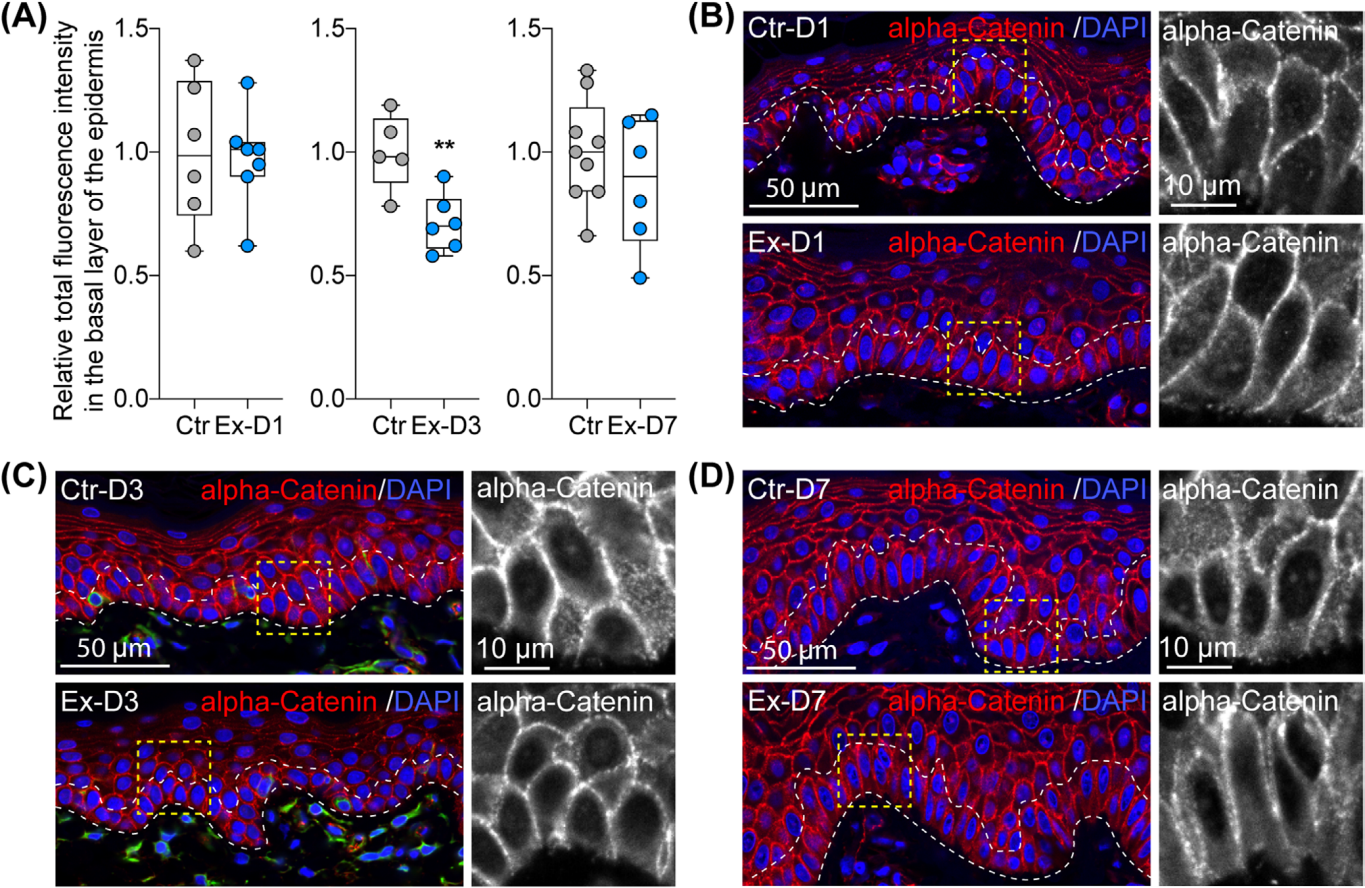
Changes in alpha‐catenin expression in skin samples during tissue expansion. (A) Quantitative analysis and (B‐D) representative images (40× magnification) of IF staining of alpha‐catenin expression in control (Ctr) and expanded skin (Ex) at day 1 (D1), day 3 (D3) and day 7 (D7) of expansion. In (A), each dot represents results for one picture. The line in the middle of the box is plotted at the median and the whiskers represent minimum and maximum values. Values represent fold changes between controls and tested samples, and the average value for each control was set as 1. Statistical significance calculated with unpaired Student *t‐*test is shown as **p ≤ 0.01. In (B‐D), dashed lines mark the basal layer of the epidermis and right panel presents the magnified views of the area marked in left panel.

IF staining of alpha‐catenin on skin samples collected at three different timepoints during tissue expansion (day 1, 3, 7) revealed significant changes in alpha‐catenin expression exclusively at day 3 (Figure 2). Specifically, the quantitative analysis showed an average 24% decrease in the basal layer of the skin expanded for 3 days compared to unexpanded control (Figure 2A presents numerical values for representative staining; FC = 0.29, *p* = 0.0061). No significant changes were observed at day 1 and day 7 of expansion, indicating that alpha‐catenin expression in the epidermis of expanded skin is not immediately and only temporally disturbed in response to mechanical stretching.

### 3.3 Nuclear Localisation of YAP1 in Basal Keratinocytes Correlates With Cell Proliferation

**FIGURE 3:**
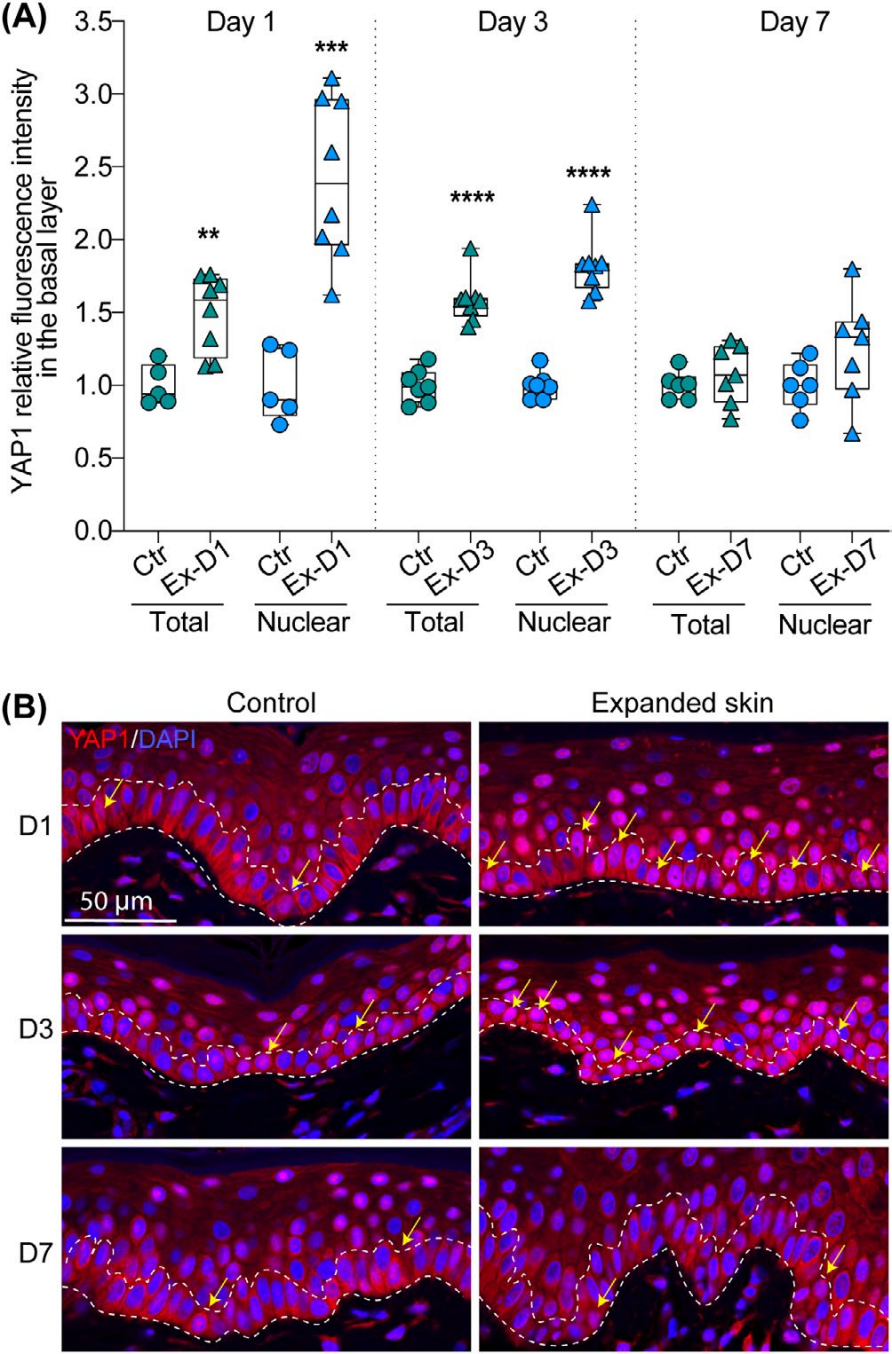
Quantification of YAP1 activation and subcellular localisation in the basal layer of the epidermis in response to mechanical stretching. (A) Quantitative analysis of YAP1 fluorescence intensity in the basal layer of the epidermis (total) and in the nuclei (nuclear) in control (Ctr) and expanded skin (Ex) at day 1 (D1), day 3 (D3) and day 7 (D7) of expansion. Each dot represents results for one picture. The line in the middle of the box is plotted at the median and the whiskers represent minimum and maximum values. Values represent fold changes between controls and tested samples, and the average value for each control was set as 1. Statistical significance calculated with unpaired Student *t*‐test is shown as **p ≤ 0.01, ***p ≤ 0.001, ****p ≤ 0.0001. (B) Representative images (40× magnification) of IF staining of YAP1 in control and expanded skin at day 1 (D1), day 3 (D3) and day 7 (D7) of expansion. Dashed lines mark the basal layer of the epidermis. The yellow arrows indicate cells with YAP1 nuclear localisation.

To investigate the effect of tissue expansion on YAP1 activation in basal keratinocytes, we examined spatiotemporal changes in YAP1 expression in the basal layer of the epidermis in skin undergoing tissue expansion (Figure 3). In control skin, YAP1 was mostly present in the cytoplasm/membrane of the basal cells, and less than 24% of basal cells showed YAP1 accumulation in the nucleus. In differentiated cells located in the suprabasal layer, the expression of YAP1 was more diffused and still concentrated in the cytoplasm. As expected, we observed a strong activation of YAP1 in basal keratinocytes under our experimental conditions. At day 1, total fluorescence intensity was significantly increased in expanded skin (Ex‐D1 group) on average by 1.38 times compared to control, indicating an early rise in YAP1 expression following expansion (Figure 3A present data for representative staining: FC = 1.50, *p* < 0.0028). Additionally, nuclear fluorescence intensity was on average 2.53 times higher in this group than in the control, suggesting stabilisation of YAP1 and its enhanced translocation from the cytoplasm to the nucleus at this early stage of expansion (Figure 3A present data for representative staining: FC = 2.42, *p* < 0.0002). By day 3, both total and nuclear fluorescence intensities remained significantly elevated in expanded skin (Ex‐D3 group) compared to control (average total: FC = 1.81**;** average nuclear: FC = 1.83), further supporting YAP1 sensitivity and activation in response to mechanical expansion (Figure 3A present data for representative staining: total FC = 1.59, *p* < 0.0001; nuclear FC = 1.80, *p* < 0.0001**)**. At day 7, no statistically significant differences were observed between the experimental and control groups for either total or nuclear fluorescence intensity (average total FC = 1.08; average nuclear FC = 1.21). This suggests that YAP1 levels normalised over time, potentially indicating cellular adaptation to tissue expansion (Figure 3A present data for representative staining: total FC = 1.08, *p* = 0.4183; nuclear FC = 1.25, *p* = 0.1526). These findings indicate that YAP1 undergoes dynamic changes during tissue expansion, as indicated by a significant increase in total and nuclear levels at days 1 and 3, followed by normalisation at day 7. These changes may reflect early mechanotransduction responses and subsequent adaptation processes in the epidermis.

**FIGURE 4:**
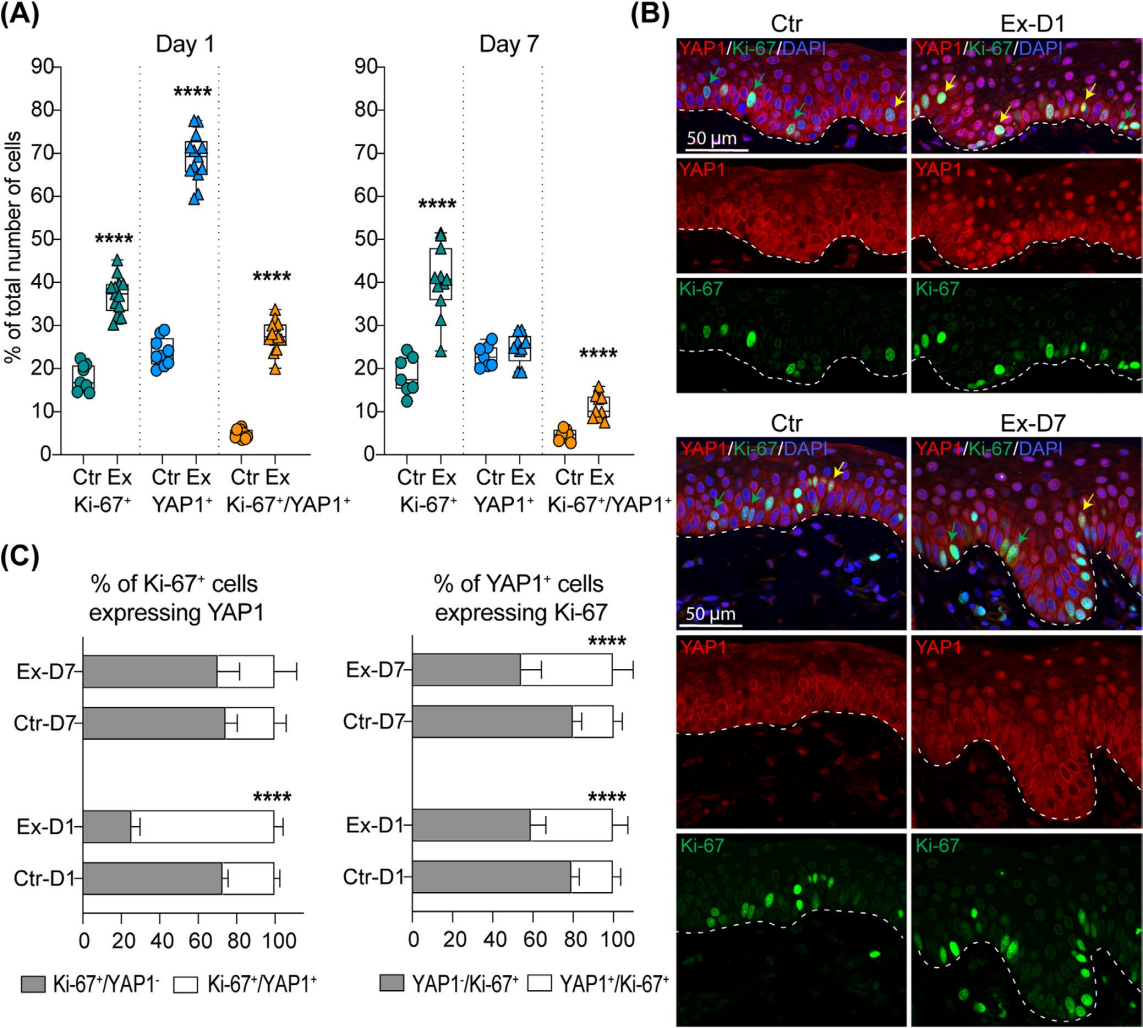
Correlation analysis between YAP1 activation and basal keratinocyte proliferation. (A) Quantitative analysis of the number of cell expressing YAP1 and Ki‐67 in the basal layer of the epidermis based on double IF staining in control (Ctr) and expanded skin (Ex) at day 1 (D1) and day 7 (D7) of expansion. Each dot represents results for one section. At least three images were analysed for each section. The line in the middle of the box is plotted at the median and the whiskers represent minimum and maximum values. Statistical significance calculated with unpaired Student *t*‐test is shown as ****p ≤ 0.0001. (B) Representative images (40× magnification) of double IF staining of YAP1 and Ki‐67 in control (Ctr) and expanded skin (Ex) at day 1 (D1) and day 7 (D7) of expansion. Dashed lines mark the epidermal‐dermal junction. The green arrows indicate Ki‐67^+^ cells, and yellow arrows indicate double positive YAP1^+^ Ki‐67^+^ cells. (C) Quantitative analysis of Ki‐67^+^ cells expressing YAP1, and YAP1^+^ cells expressing Ki‐67 in control (Ctr) and expanded skin (Ex) at day 1 (D1) and day 7 (D7) of expansion. Error bars represent SD. Statistical significance calculated with unpaired Student *t*‐test is shown as ****p ≤ 0.0001.

Further, to assess the relevance of YAP1 nuclear localisation to keratinocyte proliferation, we quantified the percentage of cells expressing Ki‐67, YAP1, and their co‐expression at day 1 and day 7 of expansion using double IF staining (Figure 4). As expected, expanded skin (Ex‐D1) exhibited a significant increase in the proportion of activated keratinocytes expressing Ki‐67 compared to control (Ctr) skin at day 1 (FC = 2.07, *p* < 0.0001). Similarly, the percentage of YAP1^+^ cells was significantly higher in the Ex‐D1 group relative to the control (FC = 2.89, *p* < 0.0001).

The population of Ki‐67^+^YAP1^+^ double‐positive cells was also elevated in the skin exposed to stretch condition compared to the control (FC = 5.74, *p* < 0.0001). In the control skin, a small fraction, on average 27%, of Ki‐67^+^ cells were also positive for YAP1 (Figure 4C). The number of proliferating cells expressing YAP1 significantly increased in expanded skin, reaching almost three times the level observed in the unexpanded control (*p* < 0.0001). The analysis revealed a positive correlation between the increase in YAP1 positive cells and cell proliferation in expanded skin (Pearson *r* = 0.90, *p* < 0.0001). However, most of the YAP1^+^ cells (80% in control and 60% in expanded skin) were not stained for Ki‐67, indicating that the presence of YAP1 in the nucleus alone is not sufficient to induce cell proliferation.

By day 7, although keratinocytes exposed to stretch still demonstrated a significantly higher proportion of proliferating cells compared to the control (FC = 2.19, *p* < 0.0001), the fraction of YAP1^+^ cells normalised to baseline (FC = 1.08, *p* < 0.0001). Simultaneously, the proportion of Ki‐67^+^YAP1^+^ double‐positive cells remained elevated in keratinocytes exposed to stretch at this time point, though to a lesser extent than at day 1 (FC = 2.47, *p* < 0.0001).

The bar graphs further illustrate the relationship between Ki‐67 and YAP1 expression at both time points. The proportion of Ki‐67^+^ cells expressing YAP1 was exclusively higher in keratinocytes at day 1 of expansion compared to control (FC = 2.76, *p* < 0.0001). Whereas the fraction of YAP1^+^ cells co‐expressing Ki‐67 was consistently elevated in the expanded epidermis across both time points (FC ≥ 1.98, *p* < 0.0001), confirming a strong association between YAP1 activation and keratinocyte proliferation under tissue expansion conditions. Together, these findings suggest that YAP1 is upregulated during tissue expansion and is closely associated with keratinocyte proliferation, indicating a potential role in mechanotransduction‐driven tissue regeneration.

Although our analysis demonstrated significant enrichment of YAP1^+^ cells expressing Ki‐67 in expanded skin at both tested time points compared to controls, the number of YAP1^+^ cells expressing Ki‐67 accounted for at most 46% of all YAP1^+^ cells. This result suggests that YAP1 activation in response to stretch is not limited to the induction of cell proliferation or that it follows different kinetics than Ki‐67 expression.

### 3.4 Human and Porcine Skin Show Pattern in Expression of Hippo Pathway Components

We stained normal human skin samples for E‐cadherin, alpha‐catenin, and YAP1 to compare the expression pattern of Hippo pathway components between porcine and human skin (Figure S2). All tested targets revealed a similar expression pattern between human and porcine skin, confirming that human and porcine skin share molecular similarities. The observed molecular similarity between porcine and human skin further implies that the findings reported here have the potential to be translated to clinical settings.

## 4 Discussion

The epidermis is a stratified, well‐organised multilayered epithelium that serves as both the first line of defence against the external environment and a bridge between the external and internal environments. One of the most fascinating features of skin is its ability to grow in response to external forces. This phenomenon is possible due to adaptive biological processes in skin such as cell proliferation [44]. In clinical settings, the ability of cells to sense and respond to changes in tissue mechanics has been adapted to stimulate skin growth through a process called tissue expansion. As previously shown by our group, tissue expansion activates complex orchestrated processes that give rise to new cells and extracellular matrix reorganisation [41]. However, the molecular mechanisms activated by mechanical forces leading to cell proliferation and skin growth during tissue expansion are not fully understood. Understanding these mechanisms may eventually lead to modification of molecular pathways for improved tissue regeneration in clinical settings. In this study, we analysed the relationship between Hippo pathway activity and cell proliferation in tissue expansion. While we did not directly measure force transmission, our findings suggest that mechanical forces applied by the tissue expander may remodel the epidermis by altering cadherin‐based AJs, potentially impacting downstream signalling pathways such as YAP1 activation. We revealed the decrease in E‐cadherin expression on mRNA and protein levels at all three tested timepoints, spanning from day 1 to day 7 of expansion, along with a simultaneous rapid YAP1 nuclear accumulation in basal keratinocytes in the expanded skin. The activation of YAP1 was not restricted to proliferating cells, raising the possibility of a broader role for YAP1 in skin adaptation to mechanical stretching. In addition, evaluation of the changes in alpha‐catenin expression at the protein level, another important component of the Hippo signalling pathway, revealed a subtle decrease in its expression that was restricted only to day 3 of expansion.

Maintaining the balance between proliferating and differentiating cells is necessary to sustain the skin's ability to grow and ensure the effective restoration of skin homoeostasis during tissue expansion [45]. In our previous work, we revealed a strong activation of cell proliferation and a local increase in basal cell differentiation in skin exposed to stretch [6, 7]. Our current study strongly suggests that the increasing demand for cell turnover and differentiation upon stretch is controlled by YAP1. Our findings are in line with previous studies indicating the role of E‐cadherin in the regulation of cell proliferation and differentation [21, 46], as well as the pivotal role of YAP1 activation in promoting cell proliferation and survival, and in control of stem cell fate [14, 15, 16, 27, 28, 30, 47, 48, 49, 50, 51, 52]. Our data suggest that E‐cadherin contributes to maintaining tissue structure under mechanical stretch; however, its role as a mechanotransducer remains to be fully elucidated. In addition, our study, conducted in a porcine model of tissue expansion, supplements previous functional studies in murine models that have identified YAP1 as a key regulator of epidermal growth in response to mechanical stimuli [27, 28]. Prior work demonstrated that genetic deletion of Yap1 in the murine epidermis significantly impaired proliferation and barrier function following mechanical stretch, while constitutive activation of YAP1 enhanced skin growth and keratinocyte proliferation during tissue expansion [27, 28]. These findings establish YAP1 as a central effector of stretch‐induced epidermal remodelling in mice. Our data extend these observations to a large animal model with skin architecture more similar to humans, supporting the relevance of YAP1 nuclear translocation in mechanically activated epidermis. By demonstrating conserved mechanosensitive responses in porcine skin, our findings strengthen the translational potential of targeting YAP1 pathways in therapeutic strategies for skin regeneration and repair.

To determine the relationship between YAP1 nuclear localisation and cell proliferation, we examined the co‐localisation of YAP1 and Ki‐67. Our study shows that temporary perturbations in the epidermal homoeostatic state during tissue expansion lead to nuclear YAP1 accumulation in basal keratinocytes, which contributes to the activation of cell proliferation and mechanically induced skin growth. Although YAP1 is thought to be primarily involved in the regulation of cell proliferation [16, 27, 28, 53, 54] and the renewal of epidermal stem cells [30, 47, 50, 51, 52], our data suggest that YAP1 may mediate other processes in mechanically induced skin growth. While the analysis of YAP1 and Ki‐67 co‐localisation in stretched skin revealed a strong association between these two proteins, we noted that the majority of YAP1^+^ cells were not Ki‐67^+^. Given that YAP1 nuclear translocation in stretched skin is not restricted to proliferating cells, we hypothesise that YAP1 activation in basal epidermal cells during tissue expansion may be involved in processes beyond cell proliferation, such as cell differentiation and survival, or that YAP1 nuclear expression follows different kinetics than Ki‐67 expression. Our results are in line with studies on cardiomyocytes exposed to acute pressure overload, showing that YAP1 acts not only as a trigger for cell proliferation but also as a pro‐hypertrophic and pro‐survival agent [55]. Similar observations have been reported in paediatric hepatocellular carcinoma tissue [56]. These findings suggest that when homoeostasis is disturbed, YAP1 plays a crucial role in diverse biological processes and is not restricted to the activation of cell proliferation. Therefore, controlling the subcellular localisation of YAP1 could play a crucial role in the transition between the proliferative and differentiating states of keratinocytes during skin growth induced by tissue expansion.

Previous research has demonstrated that in keratinocytes alpha‐catenin negatively affects YAP1 activity [15, 29]. However, we found that the decrease in alpha‐catenin protein level was delayed compared to the rapid increase in YAP1 activity in skin exposed to stretch. This suggest that during tissue expansion in large animals, YAP1 activity is primarily regulated by E‐cadherin, whereas alpha‐catenin undergoes conformational changes before its expression is affected. Evidence for tension‐driven alpha‐catenin structural changes that enable complex protein binding and downstream pathway activation, including Hippo/YAP1 pathway, have been previously described [23, 57]. Another study supporting our findings, performed on a mouse model of tissue expansion, demonstrated increased accessibility of the alpha‐catenin tension‐sensitive epitope without significant changes in alpha‐catenin expression [27].

This study adds to our knowledge of the molecular mechanisms involved in skin growth and restoration of epidermal homoeostasis during skin expansion. Using a porcine model of tissue expansion, we confirmed that external mechanical stretch applied in vitro causes increased tension on the E‐cadherin complex and releases the transcriptional activator YAP1, allowing for its translocation to the nucleus and promoting cell cycle entry. Our work emphasises the crucial role of mechanistic studies on skin growth induced by tissue expansion to design evidence‐based treatment strategies that can eventually be applied to the clinical setting. Translation of such studies to the clinical setting can improve clinical outcomes and reduce the risk of complications associated with tissue expansion.

### 4.1 Limitations

We acknowledge that the use of three biological replicates per tested condition is the gold standard for experimental studies. However, due to ethical considerations inherent to large animal research, our study was limited to six pigs across three timepoints (days 1, 3, and 7 post‐expansion). The porcine model was chosen for its close resemblance to human skin in terms of structure, physiology, and immune response, providing a clinically relevant platform for evaluating tissue expansion. Despite the limited sample size, we observed consistent activation of YAP1 at two timepoints after initiating tissue expansion. To mitigate intra‐sample variability, all immunohistochemical analyses were performed on multiple non‐consecutive sections. While the scale of the study was constrained, the findings offer meaningful insights and a strong foundation for future investigations aimed at improving clinical outcomes of tissue expansion.

### 4.2 Conclusions

We report that in a large animal model, mechanically induced basal keratinocyte proliferation is associated with changes in the E‐cadherin/Hippo/YAP1 pathway. Using a porcine model, we showed that tissue expansion alters E‐cadherin expression at the epidermal cell surface and promotes YAP1 nuclear translocation. The broad activation of YAP1 in basal cells of expanded skin is not restricted to proliferating cells, suggesting a potentially diverse role for YAP1 in the skin's adaptive response during tissue expansion.

## Author Contributions

**Joanna K. Ledwon:** conceptualization (lead), data curation (equal), formal analysis (equal), investigation (equal), methodology (lead), supervision (equal), visualization (lead), writing – original draft (lead). **Bianka Progri:** data curation (equal), formal analysis (equal), investigation (equal). **Sarah A. Applebaum:** investigation (supporting). **Oveyaa Vignesh:** investigation (supporting). **Alice Yau:** investigation (supporting). **Angie H. Aguilar:** investigation (supporting). **Adrian B. Tepole:** conceptualization (supporting), funding acquisition (equal). **Arun K. Gosain:** conceptualization (supporting), funding acquisition (equal), supervision (equal), writing – review and editing (lead).

## Ethics Statement

All animal procedures were conducted according to the protocol number IS00011674 approved by the Northwestern University Institutional Animal Care and Use Committee and in compliance with the Guide for the Care and Use of Laboratory Animals published by the National Institute of Health. Human skin tissue was collected from patients enrolled in the study STU00210182 that was approved by the Northwestern University Institutional Review Board and conducted in compliance with the Helsinki Declaration. Tissue samples were collected after obtaining written informed consent from the donors.

## Conflicts of Interest

The authors declare no conflicts of interest.

## Supporting information


**Figure S1.** Study design and timeline.

**Figure S2.** IF staining of Hippo signalling components on human skin. Representative images of IF staining on human skin. Dashed lines mark the epidermal‐dermal junction.

## Data Availability

The data that support the findings of this study are available from the corresponding author upon reasonable request.

## References

[1] C. G. Neumann , “The Expansion of an Area of Skin by Progressive Distention of a Subcutaneous Balloon; Use of the Method for Securing Skin for Subtotal Reconstruction of the Ear,” Plastic and Reconstructive Surgery 19, no. 2 (1957): 124–130, 10.1097/00006534-195702000-00004. 13419574

[2] J. LoGiudice and A. K. Gosain , “Pediatric Tissue Expansion: Indications and Complications,” Journal of Craniofacial Surgery 14, no. 6 (2003): 866–872, 10.1097/00001665-200311000-00008. 14600628

[3] A. K. Gosain , S. Y. Turin , H. Chim , and J. A. LoGiudice , “Salvaging the Unavoidable: A Review of Complications in Pediatric Tissue Expansion,” Plastic and Reconstructive Surgery 142, no. 3 (2018): 759–768, 10.1097/PRS.0000000000004650. 30148780

[4] K. E. Wooten , C. N. Ozturk , C. Ozturk , P. Laub , N. Aronoff , and R. Gurunluoglu , “Role of Tissue Expansion in Abdominal Wall Reconstruction: A Systematic Evidence‐Based Review,” Journal of Plastic, Reconstructive & Aesthetic Surgery 70, no. 6 (2017): 741–751, 10.1016/j.bjps.2017.02.018. 28356202

[5] A. M. Zollner , M. A. Holland , K. S. Honda , A. K. Gosain , and E. Kuhl , “Growth on Demand: Reviewing the Mechanobiology of Stretched Skin,” Journal of the Mechanical Behavior of Biomedical Materials 28 (2013): 495–509, 10.1016/j.jmbbm.2013.03.018. 23623569 PMC3758413

[6] J. K. Ledwon , E. E. Vaca , C. C. Huang , et al., “Langerhans Cells and SFRP2/Wnt/Beta‐Catenin Signalling Control Adaptation of Skin Epidermis to Mechanical Stretching,” Journal of Cellular and Molecular Medicine 26, no. 3 (2022): 764–775, 10.1111/jcmm.17111. 35019227 PMC8817127

[7] L. E. Janes , J. K. Ledwon , E. E. Vaca , et al., “Modeling Tissue Expansion With Isogeometric Analysis: Skin Growth and Tissue Level Changes in the Porcine Model,” Plastic and Reconstructive Surgery 146, no. 4 (2020): 792–798, 10.1097/PRS.0000000000007153. 32970001

[8] M. F. I. De La Cruz Monroy , D. M. Kalaskar , and K. G. Rauf , “Tissue Expansion Reconstruction of Head and Neck Burn Injuries in Paediatric Patients ‐ A Systematic Review,” JPRAS Open 18 (2018): 78–97, 10.1016/j.jpra.2018.10.004. 32158842 PMC7061622

[9] M. R. Yamin Ashab , N. Mozafari , M. Mozafari , and Z. Razi , “Reconstructive Surgery of Extensive Face and Neck Burn Scars Using Tissue Expanders,” World Journal of Plastic Surgery 4, no. 1 (2015): 40–49. 25606476 PMC4298864

[10] N. Bertozzi , M. Pesce , P. Santi , and E. Raposio , “Tissue Expansion for Breast Reconstruction: Methods and Techniques,” Ann Med Surg (Lond) 21 (2017): 34–44, 10.1016/j.amsu.2017.07.048. 28765784 PMC5526469

[11] M. Chiquet , A. S. Renedo , F. Huber , and M. Fluck , “How Do Fibroblasts Translate Mechanical Signals Into Changes in Extracellular Matrix Production?,” Matrix Biology 22, no. 1 (2003): 73–80, 10.1016/s0945-053x(03)00004-0. 12714044

[12] V. Vogel and M. Sheetz , “Local Force and Geometry Sensing Regulate Cell Functions,” Nature Reviews. Molecular Cell Biology 7, no. 4 (2006): 265–275, 10.1038/nrm1890. 16607289

[13] A. Tajik , Y. Zhang , F. Wei , et al., “Transcription Upregulation via Force‐Induced Direct Stretching of Chromatin,” Nature Materials 15, no. 12 (2016): 1287–1296, 10.1038/nmat4729. 27548707 PMC5121013

[14] S. Dupont , L. Morsut , M. Aragona , et al., “Role of YAP/TAZ in Mechanotransduction,” Nature 474, no. 7350 (2011): 179–183, 10.1038/nature10137. 21654799

[15] K. Schlegelmilch , M. Mohseni , O. Kirak , et al., “Yap1 Acts Downstream of α‐Catenin to Control Epidermal Proliferation,” Cell 144, no. 5 (2011): 782–795, 10.1016/j.cell.2011.02.031. 21376238 PMC3237196

[16] M. Aragona , T. Panciera , A. Manfrin , et al., “A Mechanical Checkpoint Controls Multicellular Growth Through YAP/TAZ Regulation by Actin‐Processing Factors,” Cell 154, no. 5 (2013): 1047–1059, 10.1016/j.cell.2013.07.042. 23954413

[17] B. S. Robinson and K. H. Moberg , “Cell‐Cell Junctions: Alpha‐Catenin and E‐Cadherin Help Fence in Yap1,” Current Biology 21, no. 21 (2011): R890–R892, 10.1016/j.cub.2011.09.019. 22075429 PMC5695222

[18] T. Panciera , L. Azzolin , M. Cordenonsi , and S. Piccolo , “Mechanobiology of YAP and TAZ in Physiology and Disease,” Nature Reviews. Molecular Cell Biology 18, no. 12 (2017): 758–770, 10.1038/nrm.2017.87. 28951564 PMC6192510

[19] B. D. Hoffman and A. S. Yap , “Towards a Dynamic Understanding of Cadherin‐Based Mechanobiology,” Trends in Cell Biology 25, no. 12 (2015): 803–814, 10.1016/j.tcb.2015.09.008. 26519989

[20] A. H. Huber and W. I. Weis , “The Structure of the Beta‐Catenin/E‐Cadherin Complex and the Molecular Basis of Diverse Ligand Recognition by Beta‐Catenin,” Cell 105, no. 3 (2001): 391–402, 10.1016/s0092-8674(01)00330-0. 11348595

[21] N. G. Kim , E. Koh , X. Chen , and B. M. Gumbiner , “E‐Cadherin Mediates Contact Inhibition of Proliferation Through Hippo Signaling‐Pathway Components,” Proceedings of the National Academy of Sciences of the United States of America 108, no. 29 (2011): 11930–11935, 10.1073/pnas.1103345108. 21730131 PMC3141988

[22] H. Hirata , M. Samsonov , and M. Sokabe , “Actomyosin Contractility Provokes Contact Inhibition in E‐Cadherin‐Ligated Keratinocytes,” Scientific Reports 7, no. 46326 (2017), 10.1038/srep46326. PMC5390311 28406163

[23] C. Rauskolb , S. Sun , G. Sun , Y. Pan , and K. D. Irvine , “Cytoskeletal Tension Inhibits Hippo Signaling Through an Ajuba‐Warts Complex,” Cell 158, no. 1 (2014): 143–156, 10.1016/j.cell.2014.05.035. 24995985 PMC4082802

[24] C. Ibar , E. Kirichenko , B. Keepers , E. Enners , K. Fleisch , and K. D. Irvine , “Tension‐Dependent Regulation of Mammalian Hippo Signaling Through LIMD1,” Journal of Cell Science 131, no. 5 (2018), 10.1242/jcs.214700. PMC5897721 29440237

[25] S. Dutta , S. Mana‐Capelli , M. Paramasivam , et al., “TRIP6 Inhibits Hippo Signaling in Response to Tension at Adherens Junctions,” EMBO Reports 19, no. 2 (2018): 337–350, 10.15252/embr.201744777. 29222344 PMC5797958

[26] B. W. Benham‐Pyle , B. L. Pruitt , and W. J. Nelson , “Mechanical Strain Induces E‐Cadherin‐Dependent Yap1 and Beta‐Catenin Activation to Drive Cell Cycle Entry,” Science 348, no. 6238 (2015): 1024–1027, 10.1126/science.aaa4559. 26023140 PMC4572847

[27] M. Aragona , A. Sifrim , M. Malfait , et al., “Mechanisms of Stretch‐Mediated Skin Expansion at Single‐Cell Resolution,” Nature 584, no. 7820 (2020): 268–273, 10.1038/s41586-020-2555-7. 32728211 PMC7116042

[28] Y. Xue , C. Lyu , A. Taylor , et al., “Mechanical Tension Mobilizes Lgr6(+) Epidermal Stem Cells to Drive Skin Growth,” Science Advances 8, no. 17 (2022): eabl8698, 10.1126/sciadv.abl8698. 35476447 PMC9045722

[29] M. R. Silvis , B. T. Kreger , W. H. Lien , et al., “α‐Catenin Is a Tumor Suppressor That Controls Cell Accumulation by Regulating the Localization and Activity of the Transcriptional Coactivator Yap1,” Science Signaling 4, no. 174 (2011): ra33, 10.1126/scisignal.2001823. 21610251 PMC3366274

[30] A. Beverdam , C. Claxton , X. Zhang , G. James , K. F. Harvey , and B. Key , “Yap Controls Stem/Progenitor Cell Proliferation in the Mouse Postnatal Epidermis,” Journal of Investigative Dermatology 133, no. 6 (2013): 1497–1505, 10.1038/jid.2012.430. 23190885

[31] B. Zhao , X. Ye , J. Yu , et al., “TEAD Mediates YAP‐Dependent Gene Induction and Growth Control,” Genes & Development 22, no. 14 (2008): 1962–1971, 10.1101/gad.1664408. 18579750 PMC2492741

[32] M. Ota and H. Sasaki , “Mammalian Tead Proteins Regulate Cell Proliferation and Contact Inhibition as Transcriptional Mediators of Hippo Signaling,” Development 135, no. 24 (2008): 4059–4069, 10.1242/dev.027151. 19004856

[33] E. Rognoni and G. Walko , “The Roles of YAP/TAZ and the Hippo Pathway in Healthy and Diseased Skin,” Cells 8, no. 5 (2019), 10.3390/cells8050411. PMC6562585 31058846

[34] Z. Yu , S. Liu , J. Cui , et al., “Early Histological and Ultrastructural Changes in Expanded Murine Scalp,” Ultrastructural Pathology 44, no. 1 (2020): 141–152, 10.1080/01913123.2020.1720876. 31989853

[35] P. Qiao , W. Guo , Y. Ke , et al., “Mechanical Stretch Exacerbates Psoriasis by Stimulating Keratinocyte Proliferation and Cytokine Production,” Journal of Investigative Dermatology 139, no. 7 (2019): 1470–1479, 10.1016/j.jid.2018.12.019. 30641039

[36] A. Summerfield , F. Meurens , and M. E. Ricklin , “The Immunology of the Porcine Skin and Its Value as a Model for Human Skin,” Molecular Immunology 66, no. 1 (2015): 14–21, 10.1016/j.molimm.2014.10.023. 25466611

[37] S. Debeer , J. B. Le Luduec , D. Kaiserlian , et al., “Comparative Histology and Immunohistochemistry of Porcine Versus Human Skin,” European Journal of Dermatology 23, no. 4 (2013): 456–466, 10.1684/ejd.2013.2060. 24047577

[38] J. K. Ledwon , S. A. Applebaum , B. Progri , et al., “Biological Cover Mitigates Disruption of the Dermal Structure in Mechanically Expanded Skin in a Porcine Model,” International Journal of Molecular Sciences 23, no. 21 (2022), 10.3390/ijms232113091. PMC9659138 36361876

[39] J. K. Ledwon , S. A. Applebaum , B. Progri , et al., “Acellular Dermal Matrix Cover Improves Skin Growth During Tissue Expansion by Affecting Distribution of Mechanical Forces,” Plastic and Reconstructive Surgery 153, no. 4 (2024): 663–672. 10.1097/PRS.0000000000010709 37220332

[40] T. Han , T. Lee , J. Ledwon , et al., “Bayesian Calibration of a Computational Model of Tissue Expansion Based on a Porcine Animal Model,” Acta Biomaterialia 137 (2022): 136–146, 10.1016/j.actbio.2021.10.007. 34634507 PMC8678288

[41] J. K. Ledwon , L. J. Kelsey , E. E. Vaca , and A. K. Gosain , “Transcriptomic Analysis Reveals Dynamic Molecular Changes in Skin Induced by Mechanical Forces Secondary to Tissue Expansion,” Scientific Reports 10, no. 1 (2020): 15991, 10.1038/s41598-020-71823-z. 32994433 PMC7524724

[42] K. J. Livak and T. D. Schmittgen , “Analysis of Relative Gene Expression Data Using Real‐Time Quantitative PCR and the 2(‐Delta Delta C(T)) Method,” Methods 25, no. 4 (2001): 402–408, 10.1006/meth.2001.1262. 11846609

[43] S. Ma , Z. Meng , R. Chen , and K. L. Guan , “The Hippo Pathway: Biology and Pathophysiology,” Annual Review of Biochemistry 88 (2019): 577–604, 10.1146/annurev-biochem-013118-111829. 30566373

[44] C. Blanpain and E. Fuchs , “Epidermal Homeostasis: A Balancing Act of Stem Cells in the Skin,” Nature Reviews. Molecular Cell Biology 10, no. 3 (2009): 207–217, 10.1038/nrm2636. 19209183 PMC2760218

[45] Y. Guo , Y. Song , S. Xiong , et al., “Mechanical Stretch Induced Skin Regeneration: Molecular and Cellular Mechanism in Skin Soft Tissue Expansion,” International Journal of Molecular Sciences 23, no. 17 (2022): 9622, 10.3390/ijms23179622. 36077018 PMC9455829

[46] A. J. Zhu and F. M. Watt , “Expression of a Dominant Negative Cadherin Mutant Inhibits Proliferation and Stimulates Terminal Differentiation of Human Epidermal Keratinocytes,” Journal of Cell Science 109, no. 13 (1996): 3013–3023, 10.1242/jcs.109.13.3013. 9004036

[47] T. Panciera , L. Azzolin , A. Fujimura , et al., “Induction of Expandable Tissue‐Specific Stem/Progenitor Cells Through Transient Expression of YAP/TAZ,” Cell Stem Cell 19, no. 6 (2016): 725–737, 10.1016/j.stem.2016.08.009. 27641305 PMC5145813

[48] I. Lian , J. Kim , H. Okazawa , et al., “The Role of YAP Transcription Coactivator in Regulating Stem Cell Self‐Renewal and Differentiation,” Genes & Development 24, no. 11 (2010): 1106–1118, 10.1101/gad.1903310. 20516196 PMC2878649

[49] Y. Cui , F. M. Hameed , B. Yang , et al., “Cyclic Stretching of Soft Substrates Induces Spreading and Growth,” Nature Communications 6 (2015): 6333, 10.1038/ncomms7333. PMC4346610 25704457

[50] J. H. Driskill and D. Pan , “Control of Stem Cell Renewal and Fate by YAP and TAZ,” Nature Reviews. Molecular Cell Biology 24, no. 12 (2023): 895–911, 10.1038/s41580-023-00644-5. 37626124

[51] A. Elbediwy , Z. I. Vincent‐Mistiaen , B. Spencer‐Dene , et al., “Integrin Signalling Regulates YAP and TAZ to Control Skin Homeostasis,” Development 143, no. 10 (2016): 1674–1687, 10.1242/dev.133728. 26989177 PMC4874484

[52] J. S. Mo , H. W. Park , and K. L. Guan , “The Hippo Signaling Pathway in Stem Cell Biology and Cancer,” EMBO Reports 15, no. 6 (2014): 642–656, 10.15252/embr.201438638. 24825474 PMC4197875

[53] J. Wang , Y. Zhang , Y. Gao , S. Shan , and Q. Li , “EZH2 Regulates the Correlation Between Skin Regeneration and the Duration of Mechanical Stretch,” Journal of Investigative Dermatology 141, no. 4 (2021): 894, 9–902, 10.1016/j.jid.2020.09.007. 33069730

[54] J. R. Misra and K. D. Irvine , “The Hippo Signaling Network and Its Biological Functions,” Annual Review of Genetics 52 (2018): 65–87, 10.1146/annurev-genet-120417-031621. PMC6322405 30183404

[55] J. Byun , D. P. Del Re , P. Zhai , et al., “Yes‐Associated Protein (YAP) Mediates Adaptive Cardiac Hypertrophy in Response to Pressure Overload,” Journal of Biological Chemistry 294, no. 10 (2019): 3603–3617, 10.1074/jbc.RA118.006123. 30635403 PMC6416448

[56] M. J. LaQuaglia , J. L. Grijalva , K. A. Mueller , et al., “YAP Subcellular Localization and Hippo Pathway Transcriptome Analysis in Pediatric Hepatocellular Carcinoma,” Scientific Reports 6, no. 30238 (2016), 10.1038/srep30238. PMC5015017 27605415

[57] S. Yonemura , Y. Wada , T. Watanabe , A. Nagafuchi , and M. Shibata , “Alpha‐Catenin as a Tension Transducer That Induces Adherens Junction Development,” Nature Cell Biology 12, no. 6 (2010): 533–542, 10.1038/ncb2055. 20453849

