# Supplementary figures and images for: Hippo Pathway Regulates Cell Proliferation in Skin Epidermis Exposed to Mechanical Forces
Source: J Cell Mol Med. 2025 Jun 27;29(12):e70674. doi: 10.1111/jcmm.70674 (PMC12203397; doi:10.1111/jcmm.70674)

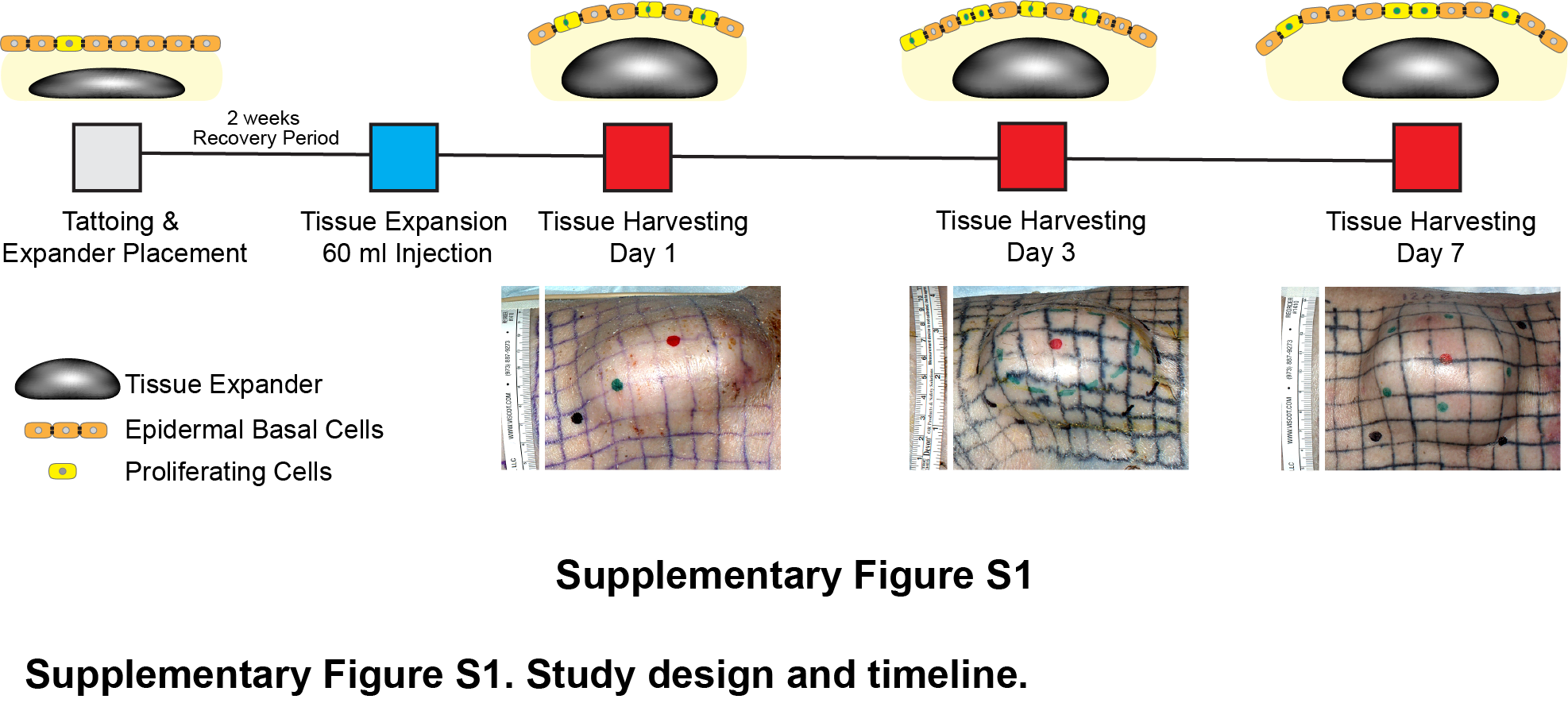

Supplement: Supplementary file 1 — Figure S1. Study design and timeline. [file JCMM-29-e70674-s001.png]

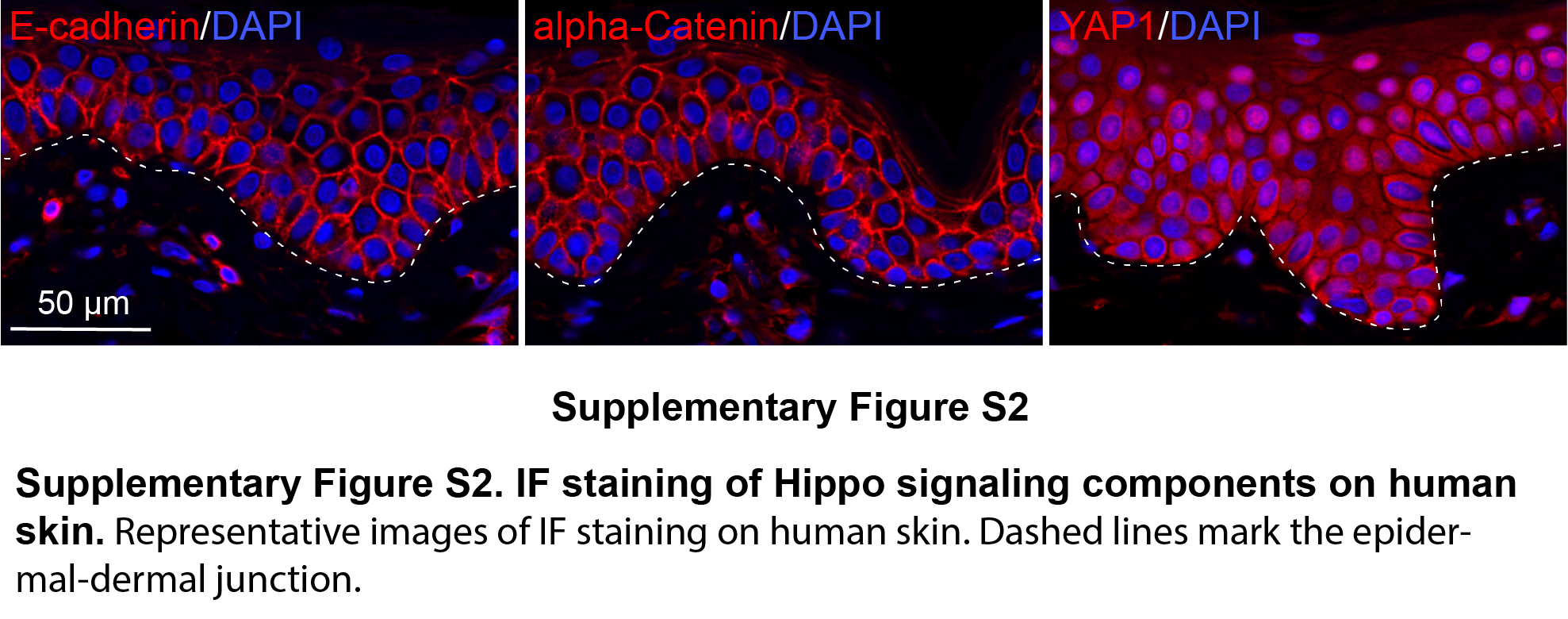

Supplement: Supplementary file 2 — Figure S2. IF staining of Hippo signalling components on human skin. Representative images of IF staining on human skin. Dashed lines mark the epidermal‐dermal junction. [file JCMM-29-e70674-s002.png]
